# CT-based radiomics for predicting radio-chemotherapy response and overall survival in nonsurgical esophageal carcinoma

**DOI:** 10.3389/fonc.2023.1219106

**Published:** 2023-08-23

**Authors:** Chao Li, Yuteng Pan, Xianghui Yang, Di Jing, Yu Chen, Chenhua Luo, Jianfeng Qiu, Yongmei Hu, Zijian Zhang, Liting Shi, Liangfang Shen, Rongrong Zhou, Shanfu Lu, Xiang Xiao, Tingyin Chen

**Affiliations:** ^1^ Department of Oncology, National Clinical Research Center for Geriatric Disorders, Xiangya Hospital, Central South University, Changsha, China; ^2^ Department of Radiation Oncology, Shenzhen People’s Hospital, The First Affiliated Hospital of Southern University of Science and Technology, Shenzhen, Guangdong, China; ^3^ Medical Science and Technology Innovation Center, Shandong First Medical University & Shandong Academy of Medical Sciences, Jinan, China; ^4^ Department of Oncology, Changsha Central Hospital, Changsha, Hunan, China; ^5^ Xiangya School of Medicine, Central South University, Hunan, Changsha, China; ^6^ Medical Engineering and Technology Research Center, Department of Radiology, Shandong First Medical University & Shandong Academy of Medical Sciences, Taian, China Technology, Shenzhen, Guangdong, China; ^7^ Perception Vision Medical Technologies Co. Ltd, Guangzhou, China; ^8^ Hunan Cancer Hospital/the Affiliated Cancer Hospital of Xiangya School of Medicine, Central South University, Hunan, Changsha, China; ^9^ Department of Network and Information Center, Xiangya Hospital, Central South University, Hunan, Changsha, China

**Keywords:** radiomics, planning ct, radiation oncology, treatment response, esophageal carcinoma

## Abstract

**Background:**

To predict treatment response and 2 years overall survival (OS) of radio-chemotherapy in patients with esophageal cancer (EC) by radiomics based on the computed tomography (CT) images.

**Methods:**

This study retrospectively collected 171 nonsurgical EC patients treated with radio-chemotherapy from Jan 2010 to Jan 2019. 80 patients were randomly divided into training (n=64) and validation (n=16) cohorts to predict the radiochemotherapy response. The models predicting treatment response were established by Lasso and logistic regression. A total of 156 patients were allocated into the training cohort (n=110), validation cohort (n=23) and test set (n=23) to predict 2-year OS. The Lasso Cox model and Cox proportional hazards model established the models predicting 2-year OS.

**Results:**

To predict the radiochemotherapy response, WFK as a radiomics feature, and clinical stages and clinical M stages (cM) as clinical features were selected to construct the clinical-radiomics model, achieving 0.78 and 0.75 AUC (area under the curve) in the training and validation sets, respectively. Furthermore, radiomics features called WFI and WGI combined with clinical features (smoking index, pathological types, cM) were the optimal predictors to predict 2-year OS. The AUC values of the clinical-radiomics model were 0.71 and 0.70 in the training set and validation set, respectively.

**Conclusions:**

This study demonstrated that planning CT-based radiomics showed the predictability of the radiochemotherapy response and 2-year OS in nonsurgical esophageal carcinoma. The predictive results prior to treatment have the potential to assist physicians in choosing the optimal therapeutic strategy to prolong overall survival.

## 1 Introduction

According to worldwide cancer statistics, esophageal carcinoma (EC) is one of the most frequent malignancies, ranking seventh in incidence and sixth in cancer-related mortality. Eastern Asia has the highest incidence and mortality, and more than 50% of these patients come from China. Esophageal squamous cell carcinoma (ESCC) comprises over 90% of all esophageal cancer cases, reflecting different treatment responses because of tumor heterogeneity (1–3). Due to the absence of typical symptoms in the early stage, EC is commonly diagnosed in the advanced stage, leading to a relatively poor prognosis (4).

Only approximately 25% of EC patients can receive radical surgery, and radiotherapy is the mainstay in the management of EC (5). The 5-year overall survival (OS) of these patients who cannot tolerate surgery due to severe heart and lung disease, hypertension, or patients who are unwilling to have surgery received radical radiotherapy varied from 20% to 73% (6). Radiotherapy is recognized as the first choice for cervical esophageal cancer because of the complex vascular nerve structure around the lesions (7). For resectable upper and middle thoracic esophageal cancer, the curative effect of radiotherapy was similar to that of surgery (8). In addition, radiotherapy is more widely applied than surgery in advanced-stage patients and early-stage patients whose lesions are adjacent to important structures (9). For these unresectable EC cases, radiotherapy with concurrent chemotherapy (CCRT) is regarded as the standard clinical workflow, significantly improving OS and decreasing treatment-related mortality (10–12). Compared to partial progressive disease (PD) or stable disease (SD) after CCRT, complete response (CR) or partial response (PR) are prone to achieve relatively high OS. Despite the great progress of radiotherapy techniques in recent decades, such as intensity-modulated radiotherapy (IMRT), volumetric modulated arc therapy (VMAT) and TOMO therapy, the CCRT response and 5-year OS rate of EC patients treated with CCRT are still not satisfactory (13, 14). It is urgent to distinguish EC patients who have the potential to benefit from CCRT in the selection of individualized strategies.

Radiomics, a critical emerging method for quantifying tumor characteristics by extracting high-throughput radiomics features from computed tomography(CT)images, magnetic resonance imaging (MRI), and positron emission tomography (PET), is now playing an important role in personalized cancer treatment (15). Previous studies explored the prediction capacity of radiomic features in many aspects of esophageal cancer, including clinical T stage, clinical N stage, lymph node metastasis, treatment response and long-term outcome of radio-chemotherapy (16–20). Some studies have also explored whether clinicopathological features and radiomics can improve EC’s prediction accuracy. Hu et al. evaluated pathologic complete response to neoadjuvant chemoradiotherapy in 231 ESCC patients using CT-based radiomics features, achieving an AUC value of 0.805 without further analyzing the predictive performance of OS (21). Philippe Lambin et al. conducted research concerning the predictive capacity of pretreatment CT radiomics in predicting 3-year OS following chemoradiotherapy with an AUC value of 0.69 in the prediction model (20). Whether CT-based radiomics and other considerable features in clinical practice, such as smoking, drinking and body mass index (BMI), can predict both treatment response and long-term outcome for EC patients with CCRT still needs further study.

Therefore, we constructed a predictive model using CT-based radiomics features in combination with clinicopathological factors to predict the CCRT response and 2-year OS in EC in this study. The selection of treatment methods may be specific due to the biological characteristics of the growth along the esophagus wall for ESCC patients, and it is clinically significant to predict the treatment efficacy before CCRT.

## 2 Materials and methods

### 2.1 Patients

This retrospective study collected 171 esophageal cancer patients treated with radiochemotherapy from January 2010 to January 2019 and was approved by the Medical Ethics Committee of Xiangya Hospital. All patients signed informed consent.

All patients were included according to the following criteria: (a) patients were diagnosed with esophageal carcinoma by histopathology; (b) age >18 years; (c) patients treated with radiotherapy and concurrent chemotherapy; and (d) the quality of radiation planning CT images was available. The exclusion criteria were as follows: (a) patients were diagnosed with other malignances and (b) patients with inferior quality of planning CT images or incomplete medical records. (c) Patients who underwent radical surgical treatment; (d) neoadjuvant chemotherapy before radiation therapy.

Besides the above standard, 80 of 171 patients used to evaluate treatment response were included according to the following criteria: (a) patients received repeated thorax CT within 1-3 months after radiotherapy and chemotherapy; (b) patients with PR were considered to be responsive, and patients with SD and PD were considered to be non-responsive. Eighty of 171 patients who met the above requirements were randomly divided into a training cohort (n=64) and a test cohort (n=16) to predict the radio-chemotherapy response.

In addition, 156 of 171 patients were randomly divided into a training cohort (n=110), validation cohort (n=23) and test cohort (n=23) to predict the 2-year survival rate. The basic clinical data were recorded and displayed (**Table 1** and **Figure S1**), including sex, age, BMI before treatment, smoking index, drinking index, pathological types, clinical stages, lesion length, clinical T stage (cT), clinical N stage (cN) and clinical M stage (cM). Smoking index is the product of the number of cigarettes smoked per day and the number of years of smoking. The drinking index is the product of daily drinking volume(ml) and years of drinking. The clinical T/N/M stages referred to the 8th TNM staging standard (2017). We recorded the date from the beginning of diagnosis to the end of death or follow-up. Patients were followed up every three months in the first year, every six months in the second year and once a year from the third to fifth year.

**Table 1:** Demographics and clinicopathological features of 133 patients in training and validation cohorts which were used to predict 2 years OS.

Parameters	Training cohort	Validation cohort	*p-*value
Gender			0.97
Male	105(95.4%)	22(95.7%)	
Female	5(4.6%)	1(4.3%)	
Age			0.42
Median(range)	59	61	
BMI			0.14
Median(range)	21.10	21.45	
Smoking index			0.55
Median(range)	400	400	
Drinking index			0.13
Median(range)	2750	5000	
Pathological type			0.90
WDSCC	35(31.8%)	9(39.1%)	
MDSCC	54(49.1%)	8(34.9%)	
PDSCC	16(14.6%)	5(21.7%)	
Others	5(4.5%)	1(4.3%)	
Clinical stages			0.01
IVB	29(26.4%)	4(17.4%)	
IVA	61(55.5%)	7(30.4%)	
III	20(18.1%)	12(52.2%)	
Lesion length			0.47
<5cm	67(60.9%)	16(69.6%)	
5-10cm	39(35.5%)	6(26.1%)	
>10cm	4(3.6%)	1(4.3%)	
Clinical T stage			0.90
T1	5(4.5%)	0(0.0%)	
T2	37(33.6%)	9(39.1%)	
T3	33(30.0%)	11(47.8%)	
T4a	17(15.5%)	2(8.7%)	
T4b	18(16.4%)	1(4.3%)	
Clinical N stage			0.42
N0	10(9.1%)	3(13.0%)	
N1	43(39.1%)	11(47.8%)	
N2	48(43.6%)	6(26.1%)	
N3	9(8.2%)	3(13.0%)	
Clinical M stage			0.41
M0	82(74.5%)	19(82.6%)	
M1	28(25.5%)	4(17.4%)	
OS			0.07
Median(days)	561	421	

### 2.2 Radiation planning CT imaging and the region of interest

Before radiotherapy, all patients underwent Siemens CT scanning (SOMATOM Definition AS). The CT scanning parameters were as follows: (a) Scanning voltages, 100-140 kVp. (b) Tube currents, 39-473 mA. (c) Exposure time, 500-1000 ms. (d) Pixel sizes of the CT images, 0.7 mm × 0.7 mm to 1 mm × 1 mm. (e) The thicknesses of slices range from 3 mm to 5 mm.

The gross tumor volume (GTV) region was delineated by two radiation physicians with 15 years of experience and reexamined by one radiologist with 30 years of experience.

### 2.3 Feature extraction

The features extracted from the region of interest (ROI) were divided into two groups: without preprocessing and after wavelet transform. A total of 1130 features were extracted for each patient with 3D-Slicer, including 14 shape features, 216 first-order features and 900 texture features. The texture features were calculated by using Gray Level Cooccurrence Matrix (GLCM), Gray Level Dependence Matrix (GLDM), Gray Level Run Length Matrix (GLRLM), Gray Level Size Zone Matrix (GLSZM) and Neighborhood Gray-tone Difference Matrix (NGTDM).

### 2.4 The prediction of the radio-chemotherapy response

A total of 80 esophageal cancer patients were divided into training and validation cohorts to evaluate treatment response. The least absolute shrinkage and selection operator (Lasso) can select variables while estimating model parameters and better solve the multicollinearity problem in regression analysis. The best predictive features were selected by using the Lasso model with 5-fold cross-validation to reduce overfitting. In addition, a logistic regression model was used to select statistically significant clinical features (p<0.1) and then establish a clinical-radiomics model to predict treatment response. The prediction ability of treatment response was evaluated by the Harrell’s concordance index (C-index) and Receiver operating characteristic (ROC) curve.

### 2.5 Prediction of 2-year OS

A total of 156 esophageal cancer patients were divided into training/test/validation cohorts to evaluate 2-year OS. The Lasso Cox regression model was used for feature selection based on the training set. We used the 10-fold cross validation method to reduce overfitting.

The optimum cutoff value was based on the value represented by the maximum specificity and sensitivity in the ROC curve. Consequently, patients were divided into a high-risk group and a low-risk group in the training set. After the survival curves of the two groups were evaluated by the Kaplan Meier (KM) method, the differences between the survival curves were tested by the log-rank test (p< 0.05).

**Figure 1:**
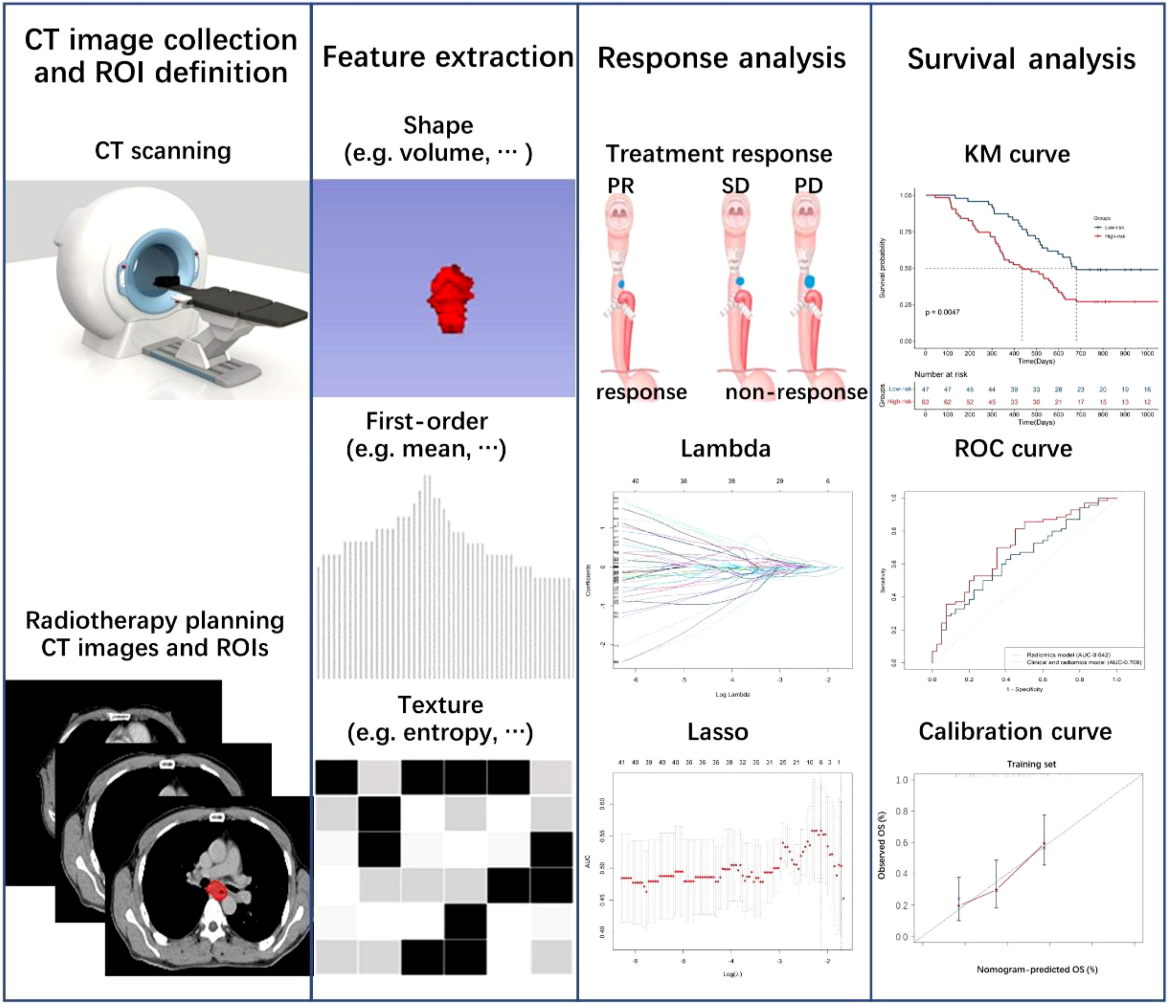
Flowchart of radiomics and clinical-radiomics model construction to predict treatment response and overall survival.

A univariate Cox proportional hazards model selected clinical features (*p*< 0.1). The selected clinical features were added into the multivariable Cox proportional hazards model based on radiomics features to improve the predictive ability. The prediction ability of the survival rate was evaluated by the C-index and ROC curve. The training set established a clinical-radiomics nomogram. Calibration curves were calculated to evaluate the consistency between the nomogram-predicted OS and recorded survival results. The flowchart of treatment response and survival model construction is presented in **Figure 1**.

### 2.6 Statistical analysis

Feature extraction was implemented in 3D-Slicer (Version 4.11). Statistical analyses were performed using R software (Version 3.4.0). The Kruskal–Wall test performed in MATLAB 2013 was used to analyze the different groups, and a p value less than 0.05 was considered statistically significant. All statistical tests were two-sided.

## 3 Results

### 3.1 Prediction of treatment response

**Figure 2:**
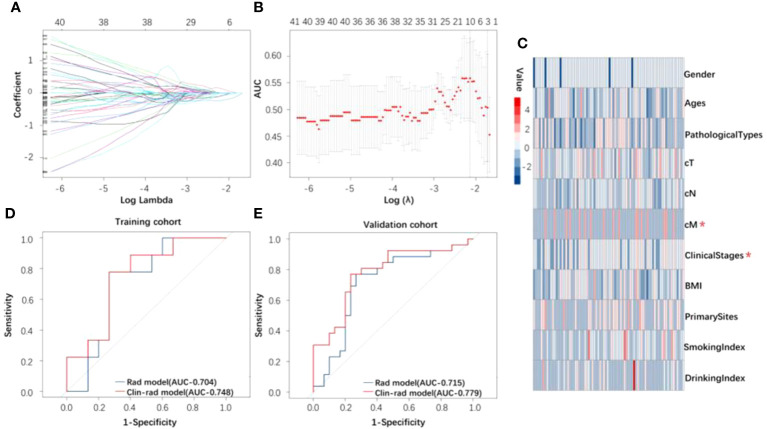
**(A)** The curve of lambda. **(B)** Lasso model used to distinguish between response and nonresponse groups. **(C)** Heatmap of all clinical features for response. The clinical features marked in the figure were those used to build the clinical and radiomics model after screening by logistic regression. **(D, E)** The ROC curve of the radiomics and clinical and radiomics model for identifying treatment response based on the training and validation cohorts.

The ROI of planning CT images was extracted from 1130 radiomics features. Wavelet-LLL-Firstorder-Kurtosis (WFK) as the best predictor of treatment response was selected by the Lasso model (**Figures 2A, B**). In this radiomics feature, there were no significant differences caused by sampling error between the cohorts used for treatment response after statistical analysis (**Table S1**). In addition, cM and clinical stages were selected from all clinical features (**Figure 2C**, **Table S2**) by logistic regression. Through the radiomics model, the AUC of the ROC curve (**Figures 2D, E**) was 0.71 and 0.70 based on the training and validation cohorts, respectively. In addition, we established a clinical-radiomics model by integrating one radiomics feature and two clinical features, the AUC of the ROC curve (**Figures 2D, E**) was 0.78 and 0.75, respectively.

### 3.2 Patient demographics and clinicopathological characteristics

A total of 156 patients were analyzed to predict 2-year OS. The clinicopathological characteristics for survival analysis (training and validation cohort) are shown in **Table 1** and **Figure S1**. In addition to clinical stages (p < 0.05), there were no significant differences between the cohorts used for survival analysis after statistical analysis to avoid sampling error.

### 3.3 Prediction of 2-year OS

Two radiomics features were selected by the Lasso Cox model as the optimal indices to predict the 2-year OS. The values of the two features were not significantly different in the training\test\validation cohorts, as shown in **Table S3**. The radiomics model was constructed by these radiomics features. A univariate Cox proportional hazards model was used to screen four clinical features, including smoking index, pathological types, cM and clinical stages. One feature called clinical stages was not included in the model because of the significant difference caused by sampling be-tween the training and validation cohorts. The analysis of clinical features is shown in **Table 2**. The clinical-radiomics model was constructed by integrating three clinical features and two radiomics features selected from above.

**Table 2:** The analysis of clinical features.

Features	HR (95%CI)	*p-*value
**Gender**	2.27 (0.56-9.26)	0.25
**Age**	1.00 (0.98-0.99)	0.71
**BMI**	1.00(1.00-1.00)	0.96
**Smoking index**	1.00(0.99-1.00)	0.06
**Drinking index**	1.00(1.00-1.00)	0.13
**Pathological types**	0.79(0.60-1.03)	0.08
**Clinical stages**	1.388(0.96-2.01)	0.08
**Lesion length**	0.97(0.65-1.46)	0.88
**cT**	0.94(0.77-1.15)	0.54
**cN**	0.85(0.62-1.12)	0.31
**cM**	1.65(0.99-2.76)	0.06

**Figure 3:**
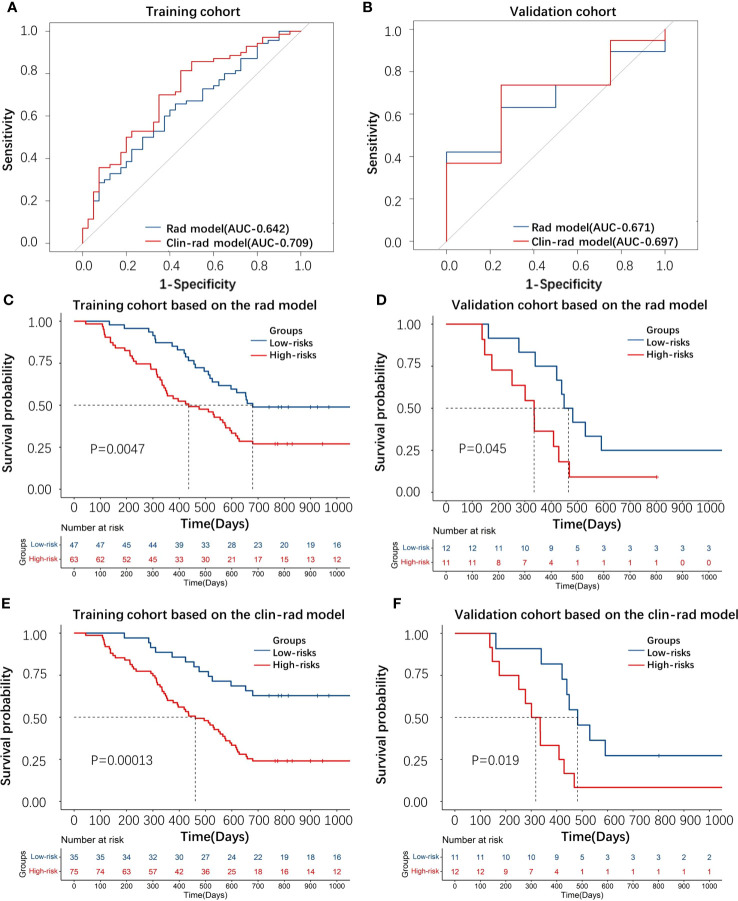
**(A, B)** The ROC curve of the radiomic and clinical-radiomics models for predicting 2-year survival based on the training and validation cohorts. **(C–F)** KM plot displaying the distinction of patients between the high-risk and low-risk groups through the radiomic and clinical-radiomics model based on the training and validation cohorts.

Through evaluating the radiomics model, the C-index of the training, test and validation cohorts was 0.62, 0.61 and 0.66, respectively, and the AUC of the ROC (**Figures 3A, B**) was 0.64, 0.60 and 0.67, respectively. In addition, by evaluating the clinical-radiomics model, the C-index of the training and validation cohorts was 0.65 and 0.68, respectively, and the AUC of the ROC (**Figures 3A, B**) was 0.71 and 0.70, respectively.

The KM curve (cutoff= 0.017 in the radiomics model and cutoff= -0.232 in the clinical-radiomics model) showed that these features distinguished the high-risk group from the low-risk group by the radiomics and clinical-radiomics models, respectively (**Figures 3C–F**).

### 3.4 The establishment of the nomogram

**Figure 4:**
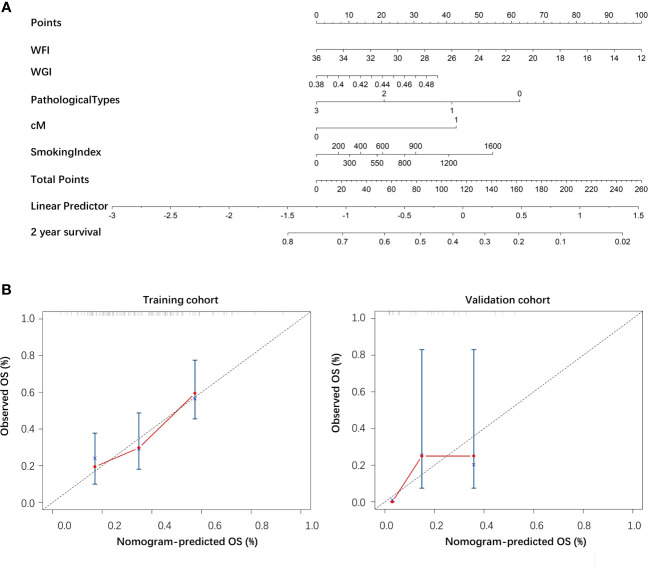
**(A)** The nomogram of the clinical-radiomics model. **(B)** The calibration curve of the nomogram based on the training and validation cohorts.

A nomogram was drawn according to the clinical-radiomics model (**Figure 4A**). Then, plotting the calibration curves of the nomogram at 2 years of OS showed that the predicted value of 2 years of OS was roughly consistent with the actual value (**Figure 4B**).

## 4 Discussion

Here, we demonstrated how radiomics features combined with clinical information to form the predictive models of EC radio-chemotherapy response and 2-year OS. EC patients with relatively poor prognoses are commonly seen in the radio-chemotherapy resistance patients because of tumor heterogeneity and the change of tumor microenvironment, which can be reflected in macroscopic images (22, 23). The concept of radiomics was developed rapidly in recent years and provides insight into ameliorating the differences of intra/inter-observers and can extract hidden information for exploring the further study (24). EC treated with CCRT-induced cells apoptosis could be used to evaluate the treatment response and prognosis according to the internal characteristics of tumor area in nonsurgical EC patients (25). Luo et al. based on the CT images of 226 patients with non-surgical esophageal cancer, radiomics and clinical features were screened out to predict CR or non-CR. Although the AUC of this model reached more than 80% in the training and validation sets, it only evaluated the short-term treatment response and did not present the long-term evaluation like the survival analysis in our study (26). Luo et al. also constructed a nomogram model for predicting local progress-free survival (LPFS) after CCRT based on radiomics and clinical features (27). Unlike the above study, we aimed to predict the overall survival rate, and the progression of the patient’s condition was not considered as the end point. In addition, Tixier et al. analyzed radiomics features of PET images from 41 esophageal cancer patients. The results showed that these features could predict the radio-chemotherapy response, and the prediction ability was higher than that of the SUV value, with a sensitivity of 76%-92% (28). In conclusion, radiomics combined with clinical features has a good predictive ability. Compared with other studies, our study predicted the short-term and long-term response of radio-chemotherapy respectively and constructed two models with good predictive performance.

In our study, we included considerable clinical features, such as BMI, drinking index and lesion length, which are important to evaluate the clinical outcomes of EC patients. Smoking and drinking are the main risk factors for male EC patients in China, and BMI is the predominant risk factor for female EC patients in China (7, 29). Compared to traditional TNM staging, our prediction model including additionally meaningful clinical features performed the better predictive ability. To predict the radio-chemotherapy response, a total of 1130 radiomic features extracted from the ROI of treatment planning CT images, such as WFK represented the best radiomic parameters. In combination with significant clinical features, our model achieved an accuracy of 75% on the validation set. The performance of the clinical-radiomic model was better than that of the radiomic model. There are several aspects could effect the results as follows. (i) The biological behavior of esophageal tumors mainly grows along with the intraluminal structure, which affects the accuracy of measuring tumor invasion. (ii) The evaluation of esophageal cancer by the RECSIT standard also has certain limitations, mainly due to the cavity structure of the esophagus and the elasticity of smooth muscle interfering with the accuracy of measurement in CT images. (iii) The uneven regression of the tumor site and the varied image quality could also disturb the accuracy.

On the other hand, 156 of 171 EC patients were involved to predict 2-year OS based on the radiomics and clinical features. Many clinical indicators such as drinking index and smoking index were included in the analysis. The calculation of the drinking index was based on Chinese spirits, which may lead to errors in the calculation of the drinking index due to the diversification of brands. The clinical T stage may not be entirely accurate due to the absence of pathological staging. The clinical N stage was only based on the number of metastatic lymph nodes and did not specifically differentiate the location of metastatic lymph nodes. Regarding BMI, recording the changes in BMI before and after treatment was more closely related to patient prognosis (30–32). WFK, WFI and WGI were all texture features extracted from radiomics, representing the essential characteristics of cancer heterogeneity (33). In the future, refining the clinical indicators included in the analysis may optimize the performance of our model and improve the robustness of the model. The model jointly constructed by radiomics and clinical indicators may be more widely used.

There are some limitations to the retrospective design of our study. (i) This study was single-center, and the prediction models constructed in this study need further validation among a larger sample size and external data in the future. (ii) More clinical parameters, such as heart disease, hypertension, diabetes and other features, should be added to our models to further improve the predictive capacity. The clinical and radiomics model with these data will be more convincing for predicting prognosis. (iii) This study did not include specific biomarkers and hematological indexes in our model to predict treatment response or overall survival. Our study further researched the relationship of radiomics with underlying molecular mechanisms.

In summary, noninvasive models based on clinicopathological characteristics and planning CT-based radiomic features had superior predictive power for tumor response and 2-year overall survival after radio-chemotherapy in esophageal cancer patients and showed greater value for translation into clinical application.

## 5 Conclusions

In conclusion, noninvasive models based on clinicopathological characteristics and planning CT-based radiomic features had superior predictive power for tumor response and 2-year overall survival after radio-chemotherapy in EC patients. These models can help clinicians make more personalized radiotherapy and chemotherapy plans and prolong the survival time of patients, which is of great clinical significance.

## Data availability statement

The original contributions presented in the study are included in the article/**Supplementary Material**. Further inquiries can be directed to the corresponding author.

## Ethics statement

The studies involving humans were approved by ethics committee of Xiangya Hospital (protocol code 202202038). The studies were conducted in accordance with the local legislation and institutional requirements. Written informed consent for participation was not required from the participants or the participants’ legal guardians/next of kin in accordance with the national legislation and institutional requirements. Written informed consent was obtained from the individual(s) for the publication of any potentially identifiable images or data included in this article.

## Author contributions

Conceptualization: DJ, TC, CL, FL, XX, and YP. Methodology: YP, JQ, YC, and YH. Software: ZZ and LTS. Validation: LFS and RZ. Resources: XY and LFS. writing—original draft preparation: CL, YP, and CHL. Writing—review and editing: DJ and TC. Supervision: LFS, RZ,YC, and TC. Funding acquisition: LFS and JQ. All authors have read and agreed to the published version of the manuscript. All authors contributed to the article.
