# Supplementary material for: CT-based radiomics for predicting radio-chemotherapy response and overall survival in nonsurgical esophageal carcinoma
Source: Front Oncol. 2023 Aug 23;13:1219106. doi: 10.3389/fonc.2023.1219106 (PMC10482418; doi:10.3389/fonc.2023.1219106)
Supplement: Supplementary file 1 [file DataSheet_1.docx]

**Appendix**

**Title:** The CT-based radiomics for predicting radio-chemotherapy response and overall survival in non-surgical esophageal carcinoma.

**Appendix A:**

**Table A1** Patient demographics and radiomics feature about treatment response analysis

| **Feature** | **Training** | **Validation** | ***p-*value** |
| --- | --- | --- | --- |
| **WFK** |  |  | 0.28 |
| Median(range) | 48.39 | 58.23 |  |

**Table A2** Demographics and clinicopathological features of 80 patients which were used to predict response

| **Parameters** | **Training** | **Validation** | ***p-*value** |
| --- | --- | --- | --- |
| **Gender** |  |  | 0.62 |
| Male | 52(92.9%) | 1(4.2%) |  |
| Female | 4(7.1%) | 23(95.8%) |  |
| **Age** |  |  | 0.41 |
| Median(range) | 61 | 59 |  |
| **BMI** |  |  | 0.95 |
| Median(range) | 21.48 | 21.59 |  |
| **Smoking index** |  |  | 0.11 |
| Median(range) | 400 | 0 |  |
| **Drinking index** |  |  | 0.31 |
| Median(range) | 3000 | 1750 |  |
| **Pathological types** |  |  | 0.80 |
| WDSCC | 21(37.5%) | 9(37.5%) |  |
| MDSCC | 23(41.1%) | 11(45.8%) |  |
| PDSCC | 8(14.3%) | 3(12.5%) |  |
| Others | 4(7.1%) | 1(4.2%) |  |
| **Clinical stages** |  |  | 0.72 |
| IVB | 14(25.0%) | 4(16.7%) |  |
| IVA | 29(51.8%) | 15(62.5%) |  |
| III | 13(23.2%) | 5(20.8%) |  |
| **Lesion length** |  |  | 0.59 |
| ＜5cm | 34(60.7%) | 14(58.3%) |  |
| 5-10cm | 21(37.5%) | 7(29.2%) |  |
| ＞10cm | 1(1.8%) | 3(12.5%) |  |
| **Clinical T stage** |  |  | 0.91 |
| T1 | 2(3.6%) | 1(4.2%) |  |
| T2 | 18(32.1%) | 9(37.5%) |  |
| T3 | 21(37.5%) | 7(29.2%) |  |
| T4a | 11(19.6%) | 4(46.7%) |  |
| T4b | 4(7.2%) | 3(12.5%) |  |
| **Clinical N stage** |  |  | 0.02 |
| N0 | 6(10.7%) | 2(8.3%) |  |
| N1 | 25(44.6%) | 5(20.8%) |  |
| N2 | 21(37.5%) | 11(45.8%) |  |
| N3 | 4(7.2%) | 6(25.0%) |  |
| **Clinical M stage** |  |  | 0.51 |
| M0 | 43(76.8%) | 20(83.3%) |  |
| M1 | 11(23.4%) | 4(16.7%) |  |

**Table A3** Patient demographics and radiomics feature about survival analysis

| **Features** | **Training** | **Test** | **Validation** | ***p-*value** |
| --- | --- | --- | --- | --- |
| **WFI** |  |  |  | 0.49 |
| Median(range) | 23.03 | 22.18 | 24.25 |  |
| **WGI** |  |  |  | 0.59 |
| Median(range) | 0.45 | 0.45 | 0.44 |  |

**Appendix B:
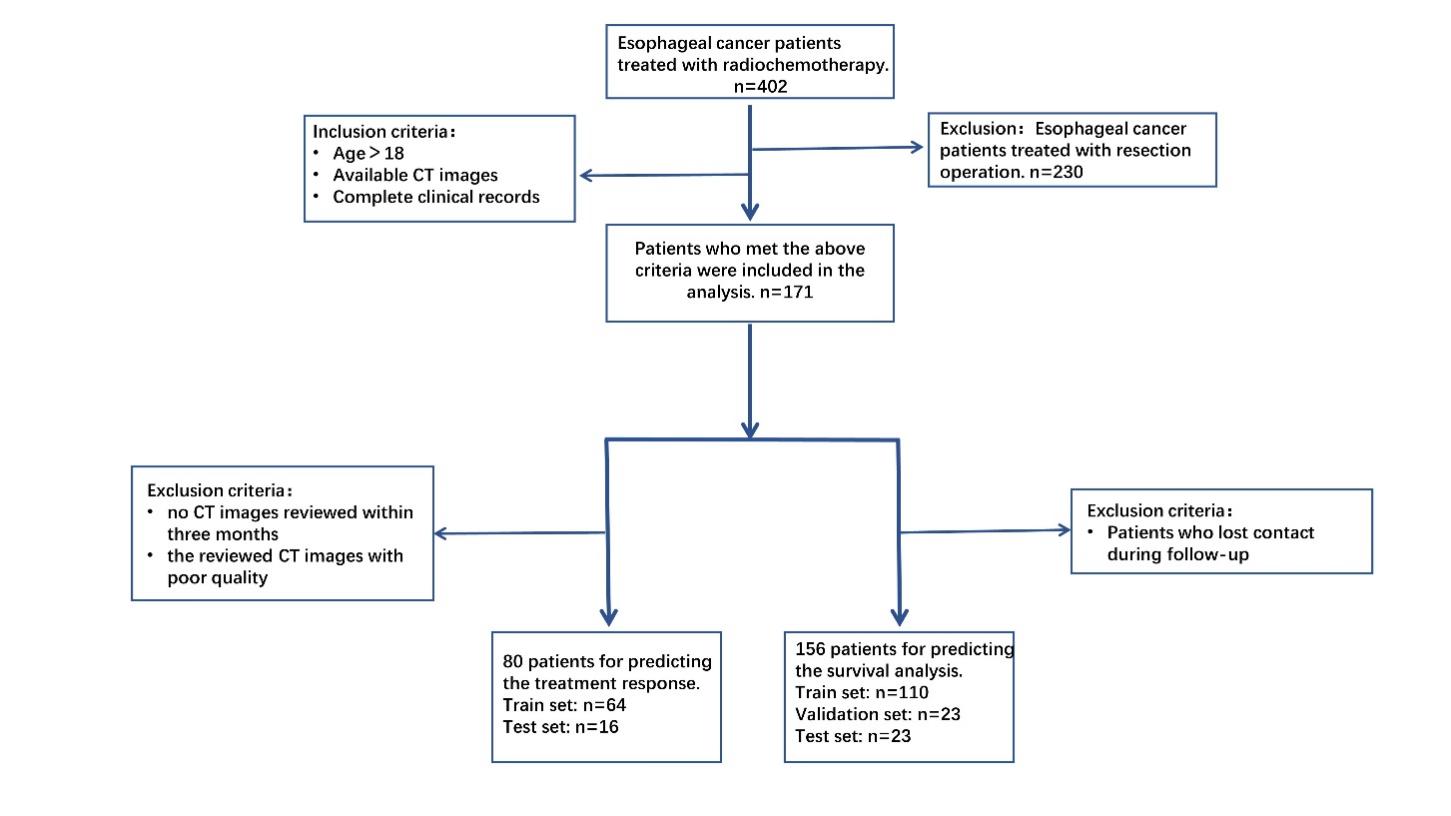
**

**Figure A1. Flow diagram showing the inclusion and exclusion criteria of EC patients.**


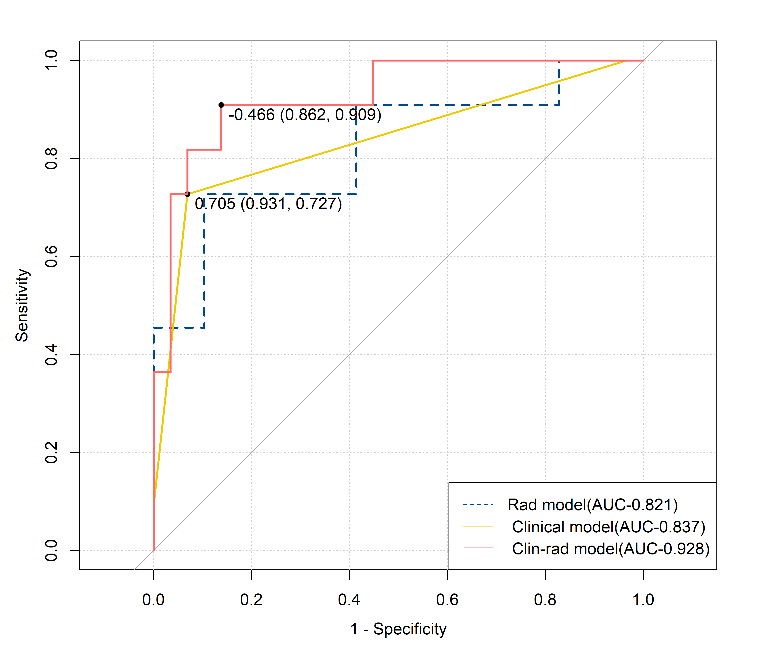
**Figure A2. The ROC of 2-year OS prediction model in the external validation set.**


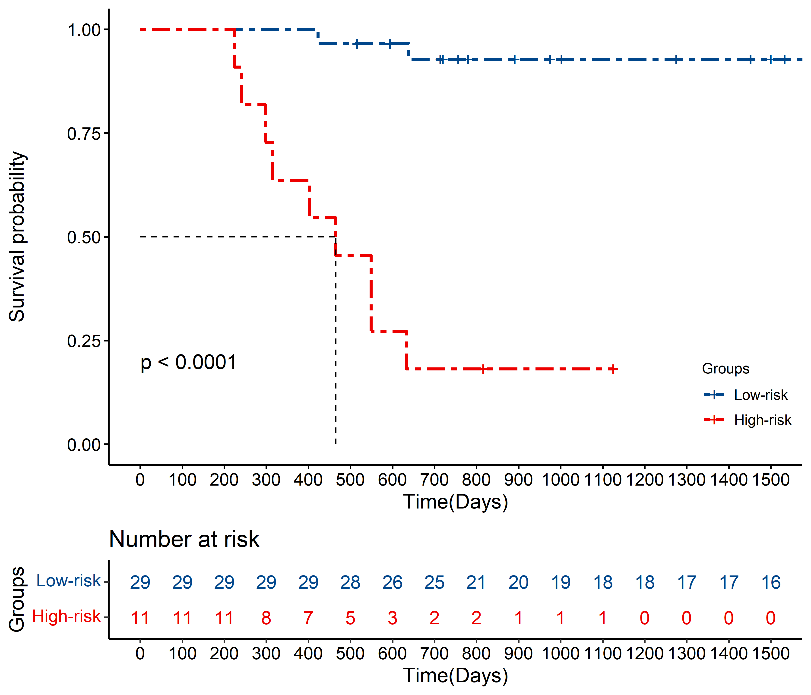


**Figure A3. The KM curve of 2-year OS prediction model in the external validation set.**


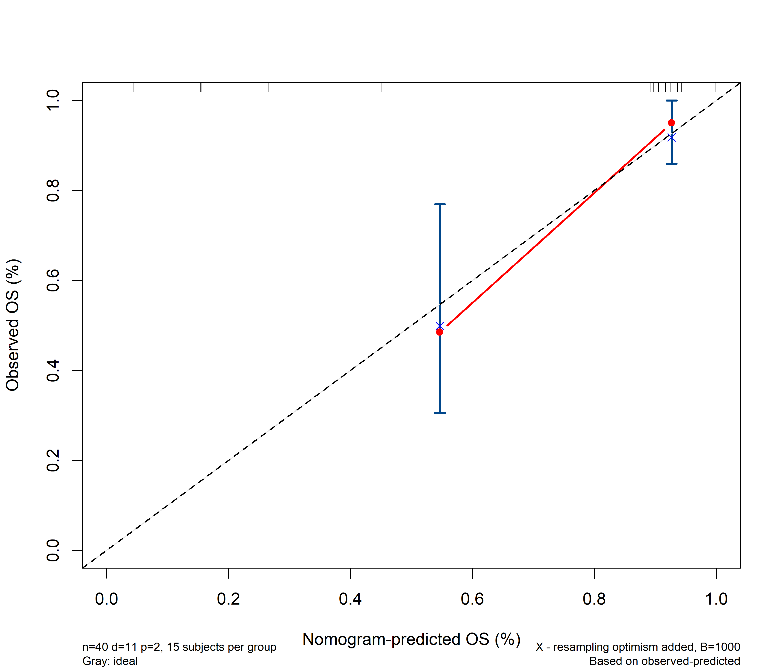
**Figure A4. The calibration curve of 2-year OS prediction model in the external validation set.**
